# Sub-micron spin-based magnetic field imaging with an organic light emitting diode

**DOI:** 10.1038/s41467-023-37090-y

**Published:** 2023-03-15

**Authors:** Rugang Geng, Adrian Mena, William J. Pappas, Dane R. McCamey

**Affiliations:** ARC Centre of Excellence in Exciton Science, School of Physics, UNSW Sydney, Sydney, NSW 2052 Australia

**Keywords:** Electronic and spintronic devices, Quantum metrology

## Abstract

Quantum sensing and imaging of magnetic fields has attracted broad interests due to its potential for high sensitivity and spatial resolution. Common systems used for quantum sensing require either optical excitation (e.g., nitrogen-vacancy centres in diamond, atomic vapor magnetometers), or cryogenic temperatures (e.g., SQUIDs, superconducting qubits), which pose challenges for chip-scale integration and commercial scalability. Here, we demonstrate an integrated organic light emitting diode (OLED) based solid-state sensor for magnetic field imaging, which employs spatially resolved magnetic resonance to provide a robust mapping of magnetic fields. By considering the monolithic OLED as an array of individual virtual sensors, we achieve sub-micron magnetic field mapping with field sensitivity of ~160 µT Hz^−1/2^ µm^−2^. Our work demonstrates a chip-scale OLED-based laser free magnetic field sensor and an approach to magnetic field mapping built on a commercially relevant and manufacturable technology.

## Abstract (web summary)

Previous demonstrations of electrically and optically detected magnetic resonance in OLED materials have established these systems as promising candidates for magnetic field sensing. Here the authors present an integrated OLED-based device for magnetic field imaging with sub-micron resolution.

## Introduction

Magnetic field sensing and mapping are important for many scientific and technological applications across both physical^1–6^ and biological systems^7–9^. Compared to classical methods, quantum sensing techniques have decisive advantages, including high sensitivity in field detection and high spatial resolution in field mapping. Among the many quantum techniques^3,10^, nitrogen-vacancy (NV) centers in diamonds have emerged as an outstanding sensor platform, and achieved room-temperature picotesla level sensitivities^11^, nanoscale spatial resolution^12–14^ and good integration and miniaturization^15,16^. Meanwhile, organic semiconductors (OSCs) are proven to be extremely sensitive to magnetic fields^17–23^, and OSC-based solid-state devices have been proposed as a new type of quantum sensor^22^. Unlike NV-based techniques, OSC-based magnetic sensors do not require optical pumping; moreover, they can provide both electrical and optical readout via electrically detected magnetic resonance (EDMR) and optically detected magnetic resonance (ODMR), respectively. EDMR allows for chip-scale integration of a single point-like sensor. Additionally, ODMR allows simultaneous acquisition of optical signals in the field of view, enabling spatially resolved sensing and imaging. OSC’s are also inherently compatible with mass-produced consumer electronics, providing a potential pathway for ubiquitous deployment.

**Fig. 1:**
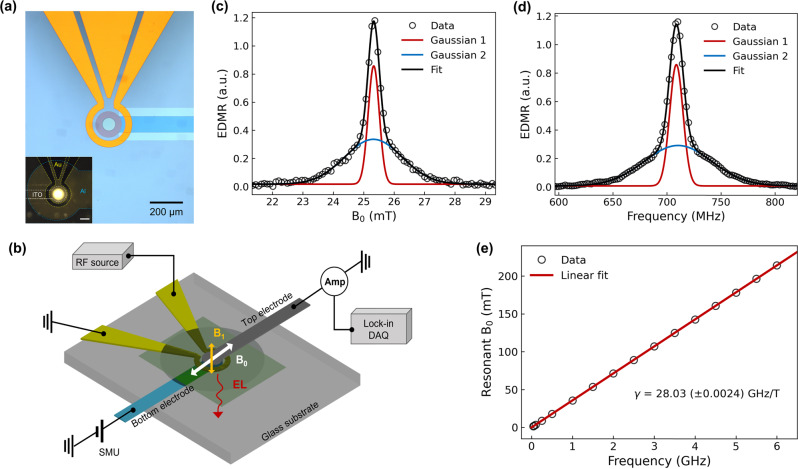
Device structure, experimental set-up, and EDMR characterization. **a** Photograph of the integrated microwave resonator where an omega-shape resonator is integrated on the prepatterned ITO/glass substrate. The active area in the middle has a diameter of 80 µm, which is defined through photolithography and insulating layer deposition. The inset shows the photograph of an integrated OLED at current of *I* = 500 nA (corresponding current density of ~10 mA/cm^2^). **b** Sketch of the integrated device structure and the experimental measurement configuration, employed with an AC magnetic field *B*_1_ created by the microwave resonator and a static magnetic field *B*_0_ generated by an external electromagnet. **c** A conventional EDMR spectrum where the static magnetic field *B*_0_ is swept with a fixed microwave frequency of 710 MHz. The spectrum is well described by the sum (black) of two Gaussian functions (red, blue), corresponding to the two hyperfine-field distributions (${\sigma }_{1}$ = 0.18(2), ${\sigma }_{2}$ = 0.94(2)) experienced by the electron and hole spins, respectively. *σ*_1_ and *σ*_2_ represent the standard deviation of the two Gaussian functions. **d** A frequency-swept EDMR spectrum where the microwave frequency is swept with a fixed magnetic field ${B}_{0}$ ≈ 25.2(5) mT via fixing the current in the electromagnet. The spectrum can be well fitted using two Gaussian functions with standard deviation of ${\sigma }_{1}\,$= 6.15(1) and ${\sigma }_{2}$ = 31.23(0), respectively. We note that the background noise caused by the frequency sweep is removed from the plots in **d**. More details are discussed in Supplementary Method 2. **e** Plot of the maximum-peak value of the magnetic field *B*_0_ in the EDMR spectrums as a function of the applied microwave frequency. A linear fit (red line) of the data yields a gyromagnetic ratio $\gamma$ = 28.03 (±0.0024) GHz/T and a corresponding $g$-factor $g$ = 2.0026 (±0.00017).

In this article, we demonstrate an integrated solid-state device to detect and image magnetic field, where a π-conjugated-polymer based organic light-emitting diode (OLED) and a microwave resonator are laterally integrated on the same substrate (Fig. 1a). This new device architecture allows one to measure the magnetic field both electrically (via EDMR) and optically (via ODMR). The device can act not only as a point sensor by measuring its bulk EDMR or ODMR response, but also as a virtual array of sensors by spatially resolving ODMR. The latter offers a route for fast magnetic mapping without point-to-point scanning, which may have potential applications in quantum magnetic sensing and imaging^4^.

The key mechanism of optically and electrically detected magnetic resonance in OLED is based on the spin-dependent recombination and dissociation dynamics of charge-carrier pairs in the active emitting layer^24^. Here we use commercial super yellow poly(phenylene-vinylene) (PPV) copolymer (SY-PPV) as the device emitting polymer layer. When positive and negative charge carriers are injected from the electrodes, they initially bind together coulombically and form electron-hole polaron pairs in the emitting polymer layer. These polaron pairs can have either singlet or triplet dominant character depending on their spin configuration, and spin mixing occurs between singlet and triplet polaron pairs via spin interaction. Polaron pairs can further recombine to form singlet or triplet excitons or dissociate back to free charge carriers with a rate which is dependent on their singlet-triplet symmetry. Under magnetic resonance ($f=\gamma {B}_{0}$, where $f$ is the microwave frequency, $\gamma$ is the gyromagnetic ratio and ${B}_{0}$ is the applied magnetic field), the spin-flips of individual charge-carriers in the polaron pairs are induced, which leads to a change of the population ratio of singlet to triplet pairs. This population change is eventually transferred to the electroluminescence (EL) and the current through the recombination and dissociation process, respectively, leading to a change in both EL and current. The induced change of the EL and the current can subsequently be detected under magnetic resonance condition.

## Results

Integrated device and EDMR characteristics. The device structure consists of two main components: an omega-shape microwave resonator, and a micron-size heterostructure OLED located at the center of the resonator (Fig. 1b). The microwave resonator is electrically isolated from the OLED using two insulating layers. See the details of the fabrication process in Method. Fig 1(a) displays the resonator-integrated substrate before the fabrication of the OLED, and the inset shows a completed device where the OLED was turned on at current $I$ = 500 nA with bright and uniform EL. We first tested the EDMR characteristics of the device. Fig 1(c) shows a typical EDMR spectrum where the microwave frequency is fixed (710 MHz) and the external magnetic field ${B}_{0}$ is swept. The EDMR signal (the change in the device current) reaches its maximum at ${B}_{0}$≈ 25.3(3) mT, matching the expected resonant frequency. The EDMR spectrum can be fit using two Gaussian functions, corresponding to the two charge-carrier species in a polaron pair^25^. See details of the EDMR measurement in the Methods. To measure the externally applied magnetic field ${B}_{0}$, the microwave frequency is swept, and the current change is monitored with lock-in detection. The change of the current is measured as a function of the microwave frequency and reaches its maximum at the resonant frequency; the external field can then be easily found given ${B}_{0}=f/\gamma$. Fig 1(d) shows a frequency-swept EDMR spectrum where the external field ${B}_{0}$ is fixed at ~25.2 mT, and the spectrum peak occurs at $f$ ≈ 708.5 MHz matching the applied magnetic field. The gyromagnetic ratio in the device is $\gamma$ = 28.03 (±0.0024) GHz/T, which is experimentally obtained through a linear fit as shown in Fig. 1(e). The value is slightly different from the free-electron gyromagnetic ratio (28.025 GHz/T) due to the weak spin-orbit coupling of the charge-carrier spin states^26,27^. In addition, the magnetic field response of the sensor is shown to be linear over more than two orders of magnitude in the frequency domain (40 MHz to 6.0 GHz). We note that the upper limit of the resonant frequency here is purely limited by the microwave source, and much higher resonance frequency can be achieved with compatible microwave sources^22,28^. The minimum field that can be directly detected here is limited by the intrinsic spin interactions (hyperfine and spin-orbit coupling) of the OSCs, although this can be overcome by applying an offset magnetic field, e.g., with a microwire^22,29,30^. In addition, for ultrasmall field detection, the influence of the earth magnetic field (~50 µT) on the spin mixing between singlet and triplet pairs needs to be considered as well^31^. The earth’s magnetic field can be shielded or compensated by applying an additional field. Microwave power broadening may also impact the EDMR spectrum^21,23^, however, we use very low power (~5 dBm) in the experiments and the effect of the power broadening on the spectrum shape is negligible. Also, the g-factor of the device is seen to remain constant, indicating that changing the microwave power to optimize the signal-to-noise ratio (SNR) will have little impact on the field detection accuracy. See more discussions in Supplementary Method 2.

### Point sensor via EDMR

**Fig. 2:**
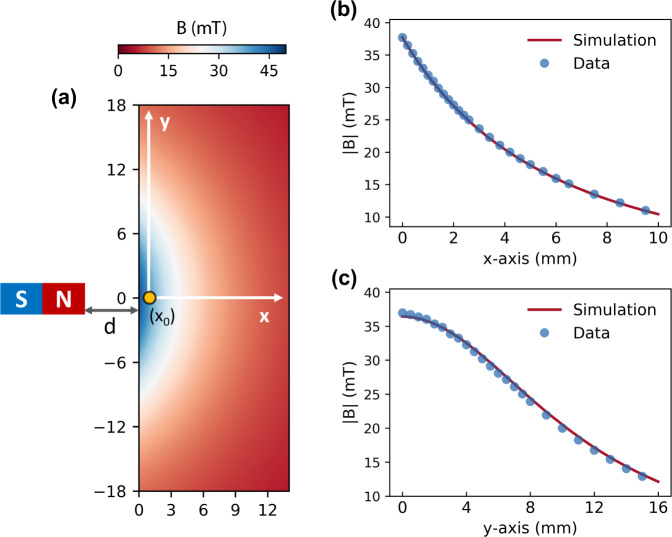
EDMR-based magnetic field sensing. **a** Sketch of the experimental set-up (not to scale). A cylindrical magnet is located next to the device with the cylindrical axis of the resulting magnetic field aligned in the plane of the device substrate. 2D simulation of the spatial distribution of the decaying magnetic field strength generated by the cylindrical magnet in a region of 14.0 × 36.0 mm in the $x-y$ plane with a distance of $d$ = 10.0 mm from the magnet. The distance *d* corresponds to the half size of the device substrate width as the OLED is located at the center of the rectangular glass substrate (see Supplementary Fig. 7). In actual experiments, we initially set a tiny gap (${x}_{0}$) between the substrate edge and the magnet at the starting position to avoid possible physical contact between them during the movement. The total distance between the OLED (yellow dot) and the magnet is $d$ +  ${x}_{0}$. The *x* and *y* coordinates represent the horizontal and vertical movement directions in the laboratory frame, respectively. The OLED here works as a point detector to measure the magnetic field strength generated by the magnet, and *x*_0_ represents the starting position of the measurement. **b** Magnetic field detection as the device is stepped along the $x$-direction. The magnetic field strength is measured via the frequency-swept EDMR spectrum at each position, and the solid curve is the simulation with an estimated starting position of ${x}_{0}$~0.20 mm. **c** Magnetic field detection as the device is stepped along the *y*-direction with an estimated starting position of ${x}_{0}$~0.40 mm.

To demonstrate the sensing capacity of the device, a permanent magnet is used to create a known imaging phantom. More details are discussed in Supplementary Method 4. Fig 2(a) sketches the experimental scheme, where the magnet is located next to the device and the OLED employed as a magnetic field point sensor via a frequency-swept EDMR measurement. We note that in OSCs the resonant peak frequency of the EDMR spectrum is only dependent on the strength of the external field ${B}_{0}$ rather than its direction^18^, and this directional-independence is also observed in our device (more details in Supplementary Method 3). Here, we carry out two independent measurements where the device is stepped along the $x$-(horizontal) and $y$-direction (vertical). At each step, the microwave frequency is swept, and the magnitude of ${B}_{0}$ determined from the resonant frequency of the resulting EDMR spectrum. The measurement results show that the sensor can detect magnetic field over a broad range with high accuracy, where the similarity between the experimental results and the computational simulations of the expected field is about 99.8% (Fig. 2b) and 98.6% (Fig. 2c), respectively. See more details in Supplementary Method 4.

### Spatially resolved ODMR and magnetic field mapping

**Fig. 3:**
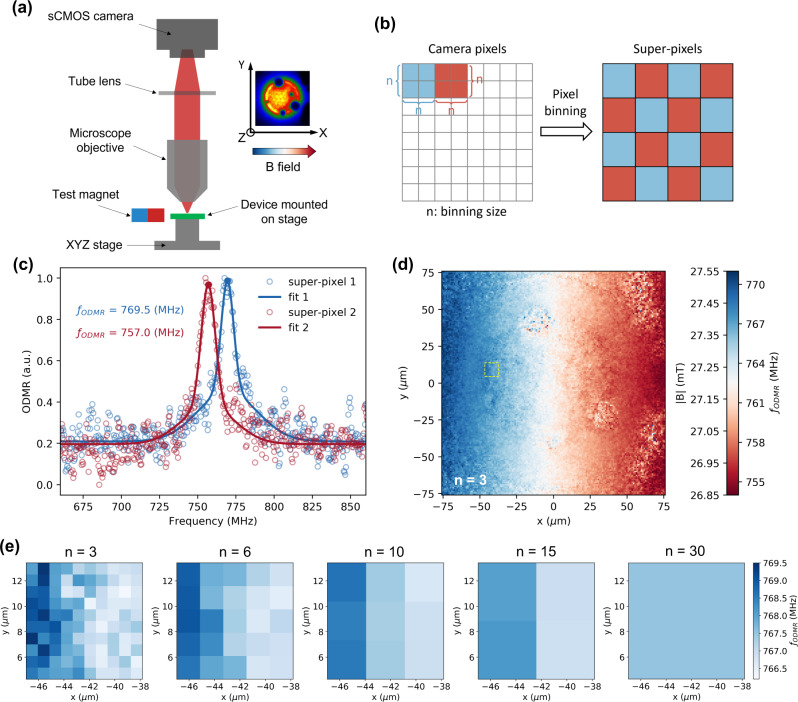
Spatially resolved ODMR-based magnetic field mapping. **a** Sketch of the set-up for spatially resolved ODMR. The inset shows the image of EL intensity captured by the sCMOS camera. The B field arrow represents the magnetic field gradient across the OLED along *x*-direction in the horizontal $x-y$ plane. **b** Scheme of pixel binning where $n$ × $n$ adjacent camera pixels are merged into one combined pixel called “super-pixel” via pixel binning process. The optical signal (EL intensity) of each super-pixel is the average of the signals of all the $n$ × $n$ individual camera pixels. **c** Double Gaussian fits of ODMR spectrums of two super-pixels with binning size $n$ = 3. Super-pixel 1 and super-pixel 2 corresponds to the super-pixel at position of (−63.4 µm, 0.0 µm) and (52.4 µm, 0.0 µm) in **d**, respectively. The solid circle dots label out the resonant peak position in the fit curves. **d** 2D spatial map of the resonance frequency (${f}_{{{{{{\rm{ODMR}}}}}}}$) of the ODMR spectrums of 166 × 166 super-pixels with binning size $n$ = 3. The entire region contains 500 × 500 camera pixels, and the super-pixel size is about 0.91(5) × 0.91(5) µm ($n$ = 3). Weak EL signal is also observed outside the defined area of the OLED due to the high hole conductivity of the PEDOT:PSS thin film. This provides the ODMR spectrums across the entire region. **e** The most left figure ($n$ = 3) shows a zoom-in view of a sub-region (10 × 10 super-pixels) of the 2D map in **d**, which is marked by the yellow dash square in **d**. The $x-y$ coordinates in **e** are consistent with that in **d**. The rest four figures in **e** show the spatial map of the magnetic field in the same sub-region but with different binning sizes.

This integrated device is not only capable of sensing magnetic fields electrically via EDMR, but also provides optical accessibility for magnetic field mapping via a spatially resolved ODMR. To measure the spatially resolved ODMR, an optical microscope is used to image the device onto an sCMOS camera (Fig. 3a). A square-wave microwave signal (0.5 Hz) is applied, and the difference in EL between the on and off cycles is measured with the camera. The microwave frequency is swept, and a similar image is taken at each frequency. Each pixel of the camera therefore measures an ODMR spectrum associated with a spatial region of the OLED. See more details in the Methods. To improve the SNR of the ODMR spectrum so that the magnetic field can be more precisely measured, the camera pixels are binned to form super-pixels (see Fig. 3b). Fig 3(c) shows the ODMR spectrums of two individual super-pixels at separate locations. Though the SNR of the data is still relatively low (~4) after binning, the spectrum can be well fit using a double Gaussian function, from where the magnetic field is acquired by converting the resonant frequency (${f}_{{{{{{\rm{ODMR}}}}}}}$) to a field strength (|${{{{{\bf{B}}}}}}$|). Fig 3(d) shows a 2D map of the measured magnetic field across an entire region (152.5 × 152.5 µm) with super-pixel size of ~0.91 µm (binning size $n$ = 3). The super-pixel size here is above the optical diffraction limit of the microscope objective ($\lambda /(2{{{{{\rm{NA}}}}}})$ = 714 nm) for a typical EL wavelength of $\lambda$ = 600 nm. We note that the optical diffraction will set a threshold of the spatial resolution in our sensor, as well as in other alternative techniques (i.e., NV centers in diamonds) where a digital camera is used for the simultaneous acquisition of the spatially resolved optical readout in the field of the view.

The measured magnetic field shows a clear and smooth gradient change along the *x*-direction while remaining the same along the *y*-direction, which is consistent with the orientation of the magnet. We note that the subtle ring feature present in the center of the 2D field map is caused by the dielectric layer that defines the active region of the OLED in the photolithography process (see Supplementary Method 1). The EL signal and the related ODMR spectrum is observed across the whole field of the view, which is much larger than the OLED region ($D$ = 80 µm) (see Supplementary Fig. 1f). The reason is because of the high hole conductivity (>1000 S cm^−1^)^32–34^ in the layer of poly(styrene-sulfonate)-doped poly(3,4-ethylenedioxythiophene) (PEDOT:PSS). Consequently, the injected holes from the indium tin oxide (ITO) electrode diffuse in the PEDOT:PSS layer along the in-plane direction, leading to a EL emission over a much larger area. The SNR of the ODMR spectrums differ inside and outside of this region (see Supplementary Fig. 9). Fig 3(e) shows a zoom-in view of a local region (9.1 × 9.1 µm) of the 2D map in Fig. 3(d) with a variety of binning size. As the binning size increases, the spatial resolution of the field mapping decreases; accordingly, the standard error of the fit decreases, indicating the enhancement of the measurement sensitivity. See more details in Supplementary Fig. 9.

We now turn to the relationship between the magnetic field sensitivity and the spatial resolution of the field mapping. In general, the magnetic field sensitivity is defined as the minimum detectable magnetic field difference ${\delta B}_{\min }$, which corresponds to the measurement error. By combining the signal from neighboring pixels, the measurement error can be reduced by $\sqrt{N}=n$ times, where $N$ is the total number of camera pixels in each individual super-pixel with binning size $n$ ($N=n\times n$), although this also reduces the spatial resolution. To account for the measurement time, the sensitivity is represented as ${\eta=\delta B}_{\min }\times \sqrt{T}={{{{{{\mathrm{SE}}}}}}}(n)\times \sqrt{T}$, where ${{{{{{\mathrm{SE}}}}}}}(n)$ is the standard error (${{{{{\rm{SE}}}}}}$) of a single ODMR measurement of an individual super-pixel with binning size $n$, and $T$ is the total data acquisition time for each microwave frequency step. We note that $T$ = 400 s is used in the actual experiment (see Supplementary Method 6), and the ${{{{{\rm{SE}}}}}}$ of measurement can be extracted from the fit of the ODMR spectrum. For magnetic field mapping with spatial resolution of ~0.91(5) µm (binning size $n$ = 3) in Fig. 3(d), the magnetic field sensitivity is ~233.04 µT Hz^−1/2^ in the OLED region and ~163.16 µT Hz^−1/2^ in the diffusion region. When the super-pixel size is increased to ~14.64 µm (binning size $n$ = 48), the sensitivity is improved to ~136.88 µT Hz^−1/2^ in the OLED region and ~40.75 µT Hz^−1/2^ in the diffusion region. Although the measured sensitivity follows the general $1/n$ rule (see Supplementary Fig. 12), the actual improvement ratio of the sensitivity (e.g., 163.16 ÷ 40.75 ≈ 4 in diffusion region) is much smaller than the trade-off ratio of the spatial resolution 48 ÷ 3 = 16 times). We suspect that this discrepancy arises due to device-related noises including the fluctuation of EL intensity caused by the electrical coupling between the OLED and the resonator (see Supplementary Fig. 4), and other technical contributions, which suppress the SNR improvement obtained via binning. The continuous wave (CW) ODMR shot-noise-limited sensitivity of our current set-up is estimated to be 54.8 µT Hz^−1/2^ µm^−2^, which is about three times better than the measured sensitivity in this work. Details of the calculation can be found in the Methods.

**Fig. 4:**
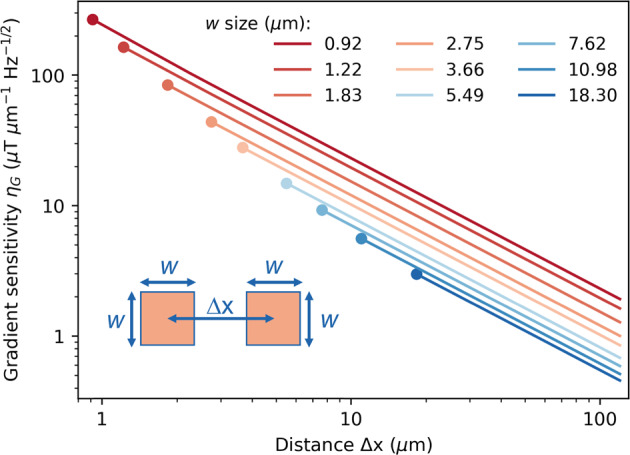
Magnetic field gradient sensitivity. The inset shows two virtual point sensors with given size of $w\times w$ and gap distance of $\triangle x(\triangle x\ge w)$. The dots at the left end of each curve represent the starting position where $\triangle x=w$, indicating the spatial resolution limit of the field gradient. The figure is in log–log scale plot.

Alongside magnetic field mapping, the device can also be used to measure magnetic field gradients at µm scales. The field gradient is defined as $G=\triangle B/\triangle x$, where $\triangle B$ is the difference of the measured magnetic field between two super-pixels with size of $w\times w$ acting as two virtual point sensors, and $\triangle x$ is the center-to-center distance between them. The averaged gradient along the *x*-direction in Fig. 3(d) is estimated as ~3.7 µT/µm ($\triangle B$ ≈ 555.7 µT, $\triangle x$ ≈ 151.0 µm), while no clear gradient is observed along the *y*-direction due to the device alignment relative to the orientation of the magnet (see Fig. 2a). The minimum distinguishable field difference between two neighboring points ${\left(\triangle B\right)}_{{{\min }}}$ is determined by the magnetic field sensitivity at individual point (the minimum detectable magnetic field difference ${\delta B}_{\min }$), which is dramatically impacted by the binning size; therefore, the field gradient sensitivity (or the minimum detectable field gradient difference) is limited by the size of the virtual point sensors. In addition, the size of the virtual point sensors also sets a limit on the spatial resolution (${(\triangle x)}_{{{\min }}}=w$) of the field gradient. Based on error propagation, the magnetic field gradient sensitivity ${\eta }_{G}$ can be calculated as a function of virtual pixel size $w$ and the gap distance $\triangle x$. For details of the calculation refer to Supplementary Method 6. As shown in Fig. 4, the field gradient can be achieved at µm scale with a relatively good gradient sensitivity. The gradient sensitivity can be improved by either increasing the virtual pixel size, the gap distance or increasing both at the cost of spatial resolution. We note that all the noise sources that limit the magnetic field sensitivity discussed above will limit the gradient sensitivity as well.

## Discussion

One significant challenge of using any resonance-based technique for magnetic field sensing is the measurement time to find the resonant frequency across a broad range, which can be time-consuming, particularly with small frequency step size and long averaging time for better SNR. We anticipate that operationally a coarse scan across a broad range followed by a fine scan across the resonance range can be used to shorten the measurement time. However, such an improvement may be limited by the resonance linewidth, which sets the upper limit of the frequency step size. Another challenge is the field sensitivity in both EDMR and ODMR, especially in the spatially resolved ODMR where the SNR is much lower compared to the bulk counterpart. This can be improved in a number of ways: firstly, by minimizing the sensor-related noises, especially the electrical coupling between the resonator and the OLED through device architecture optimization. Secondly, the sensitivity can be further enhanced by using techniques, which exploit coherent quantum effects (e.g., Ramsey or other dynamical decoupling schemes)^14,35–38^. One of the motivations for this study is the presence of reasonably long spin phase coherence times of polarons in organic devices at room temperature. Spin phase coherence times (*T*_2_) approaching 1 µs at room temperature has been observed in EDMR measurements^39,40^, and perdeuterated organic materials can be used to further increase these times^18,41^. Concerted efforts aimed at identifying or developing materials with even longer phase coherence times appear to be promising.

In contrast to NV-based detections where multiple resonant peaks may occur because of the different crystallographic axes of NV in diamond, there is only one resonant peak in our sensor, sensitive to the strength of the external magnetic field regardless of the field orientation. This means that no alignment of the sensor is required in the detection of the field strength. This alignment-free characteristic can potentially be useful in applications where the magnitude rather than the direction of the field is of importance, and inaccuracy caused by improper orientation of the magnetometer can be reduced (i.e., Hall-effect probe). We note that the amplitude of the resonant signal is proportional to the projection of the microwave field ${B}_{1}$ along the static external field ${B}_{0}$, and it reaches the minimum or even vanishes when ${B}_{0}$ is in parallel with ${B}_{1}$. More details are discussed in Supplementary Method 3. The sensitivity issues caused by this can be potentially solved by generating a ${B}_{1}$ field with large directional inhomogeneity across the OLED so that there is always a portion of ${B}_{1}$ field that is projected orthogonal to *B*_0_, leading to measurable resonances regardless of the orientation of ${B}_{0}$.

In order to detect the direction of the magnetic field, we can potentially extend the device architecture with two mutually perpendicular metallic strip lines integrated underneath the OLED^22^. This will provide an in-plane microwave field with arbitrary direction. Along with the out-of-plane microwave field from the already integrated resonator, there will be three independent microwave fields that are perpendicular to each other. By repeating the measurement with each microwave field, the corresponding vector components of the unknown magnetic field can be detected. We note that the use of the metallic strip lines may block the light emission and consequently limit the optical readout in ODMR. Additionally, these metallic layers including the top Al electrode of the device can distort the external magnetic field^28^, posing more challenge for the precise vector measurement, especially at high magnetic fields.

It’s worth nothing that our device could potentially detect complex magnetic objects (e.g., arrays of magnetic elements, ferromagnetic surfaces). Depending on the characteristic feature size of the magnetic object and the spatial resolution required, the proximity between the active layer of the device and the surface of the object is typically in the µm range or even smaller. Since the typical thickness of commercial device substrates (e.g., quartz or other dielectric materials) measures at approximately 50 µm or more, the best way to achieve the required proximity is to place the object to be imaged directly upon the top electrode. Although the top electrode is protected by a pre-coated cap layer, both components are very thin (~10’s of nm), which should enable imaging features on these length scales.

Compared to established alternatives for magnetic field sensing (e.g., NV in diamond, SQUIDs, Hall-effect), our OLED-based device is laser-free, room-temperature, and capable of both optical and electrical readout. In addition, our device is designed to be compatible with commercially available OLED technologies, providing the unique ability to map magnetic field over a large area or even a curved surface. While our study demonstrates a clear technology pathway, more work will be required to increase the sensitivity and readout times. Nonetheless, the trade-off between cheap manufacturability and sensitivity may make this approach suitable for a range of applications where ubiquitous sensing is valued over absolute sensitivity.

## Methods

### Fabrication of the integrated microwave resonator

To have optical access to the device, the resonator structure and the OLED should be laterally separated so that the light can emit out from the ITO/glass side of the substrate. The main challenge of integrating a resonator with an OLED on the same ITO-based glass substrate is how to electrically isolate them from each other. Here, we employ low-temperature atomic-layer-deposition (ALD) method for the insulating layer deposition, providing conformal and high-quality electrically insulating layers with thin thickness. The main procedures are as follows: (1) prepatterned ITO (120 nm) on glass substrates (30.0 × 20.0 × 0.7 mm) was purchased from a commercial company. (2) prepare the first insulating layer Al_2_O_3_ between the ITO layer and the following resonator layer. The geometry of the insulating layer was patterned through standard photolithography process (MA6 system with negative photoresist nLOF2020 and developer ZA826MIF), and the Al_2_O_3_ (45 nm) layer was deposited by low-temperature ALD, followed by the lift-off process in NMP bath. (3) prepare the resonator layer on top of the first insulating layer. The structure of the resonator was defined through standard photolithography process (the same as in step 2), and then metal layer of Ti (10 nm)/Au (500 nm)/Ti (10 nm) was thermally deposited in a thermal deposition chamber (Jurt J. Lesker) followed by standard lift-off process. The 10 nm Ti layers were adhesion layers. (4) prepare the second insulating layer on top of the resonator the same way as for the first insulating layer in step 2. This second insulating layer of Al_2_O_3_ (45 nm) is to electrically isolate the resonator itself from the top electrode of the OLED, which was deposited in the later device fabrication process. The final layer structure of the resonator-integrated substrate is: bottom electrode layer of ITO (120 nm)/first insulating layer of Al_2_O_3_ (45 nm)/microwave resonator layer of Ti (10 nm)/Au (500 nm)/Ti (10 nm)/second insulating layer of Al_2_O_3_ (45 nm). More details of the fabrication process are discussed in Supplementary Method 1.

### Fabrication of micron-size OLED onto the resonator-integrated substrate

The resonator-integrated substrate was firstly cleaned by using UV ozone cleaner (purchased from Ossila) for 10 min, followed by spin coating of PEDOT: PSS (purchased from Heraeus, Al 4083) at 3000 rpm for 1 min. The PEDOT:PSS thin film was baked for 2 h at 120 °C on hotplate, resulting in a film thickness of about 35 nm. The sample was then transferred to a glove box (O_2_ < 0.5 ppm, H_2_O < 0.5 ppm) where the SY-PPV solution (3 mg/ml in toluene) was spin coated at 1200 rpm for 1 min followed by a post bake for 2 h at 60 °C on hotplate, resulting in a film thickness of about 80 nm. Before the spin coating, the SY-PPV solution was filtered using a PTFE syringe filters with pore size of 0.45 μm to remove the polymer aggregates. The extra part of the SY-PPV layer on the top of the Au resonator and the electrode pads was carefully removed by using cotton rod. Then sample was transferred to a high vacuum chamber (<10^−8^ mbar) for the deposition of LiF (1 nm)/Al (100 nm) using a shadow mask. The shadow mask was carefully aligned with the substrate so that Al was deposited onto the target area only (on top of the second insulating layer region), which is to avoid any possible short-circuit connection between the resonator and the top Al electrode. After the fabrication, the device was encapsulated with a thin glass lid with recessed cavity using UV-activated epoxy inside the glove box. A thin desiccant sheet (as moisture and oxygen absorber) was sticked onto the inner surface of the recessed cavity to prevent the device degradation from the air.

### EDMR measurement set-up

For the EDMR measurements in Figs. 1 and 2, the OLED was operated under a constant current of 0.5 µA (Keysight, SMU B2901A) at room temperature. The device was mounted onto a PCB with electrical connection via pogo pins, and the PCB was connected to the measurement instruments using SMA cables. For details of the set-up refer to Supplementary Fig. 7. A signal generator (SRS SG396) was connected to the microwave resonator with 5 dBm power output, which was pulse modulated with 10 μs pulse width and 10 kHz modulation. The other end of the resonator was connected to a 50 Ω terminator. During the EDMR measurement, the resulting periodic change of the device current was first amplified by a low-noise current amplifier (SRS SR570) with a 6 dB bandpass filter at 10 kHz, and then detected by a lock-in amplifier (SRS SR865A).

### Spatially resolved ODMR measurement

As shown in Fig. 3(a), the device was mounted on a 3-axis optical stage and well aligned with an optical imaging system. The light emitting out of the device from the ITO side, is collected by an Infinity Corrected objective (20x Mitutoyo Plan-Apochromat Objective, NA = 0.42, working distance = 20.0 mm, focus length = 10.00 mm), and then refocused onto a scientific CMOS camera (Andor iStar sCMOS 18U-A3 with working temperature of 0.0 °C) through a compatible tube lens (focal length = 200.0 mm), where the EL intensity signal was detected and acquired by the camera. Under 20x magnification, the pitch between two adjacent pixels on the OLED plane is about 0.30(5) µm. The OLED was operated under a constant current of 0.5 µA (Keysight, SMU B2901A) at room temperature, and the resonator was connected to the signal generator (SRS SG396, 5 dBm power output). A test magnet was located next to the device, providing a static external magnetic field for Zeeman energy splitting. The microwave singal was modulated by a 0.5 Hz square-wave sequence with 200 operation sequences, and the EL intensity signal in the on and off cycle was recorded by the camera (exposure time of 980 ms) at each microwave frequency. The microwave frequency was swept, and eventually a full set of EL intensity data was recorded as a function of the microwave frequency. By calculating the averaged change of the EL signal between on and off cycle as a function of microwave frequency, we were able to obtain the ODMR spectrum at each camera pixel, namely spatially resolved ODMR spectrum.

### Shot-noise-limited sensitivity

CW ODMR shot-noise-limited sensitivity is calculated in the following equation^35^: ${\eta }_{{{{{{\rm{CW}}}}}}-{{{{{\rm{ODMR}}}}}}}=\frac{8\pi }{3\sqrt{3}}\frac{\hslash }{{g}_{e}{\mu }_{B}}\frac{\varDelta v}{C\sqrt{R}}$, where $C$ is the contrast of the ODMR spectrum, $R$ is the effective rate of photon detection of the OLED emission per µm^2^, and $\Delta v$ is the linewidth of the ODMR resonance. In our measurement, the contrast $C$ varies between 0.23% to 0.55% depending on the location. The camera pixel well depth $P$ is 3 × 10^4^ electrons, the exposure time ${\tau }_{\exp }$ ~ 1.0 s for the maximum raw EL signal, and the peak quantum efficiency (QE) of the camera is about 50%. Given the individual pixel size $A$ (0.31 × 0.31 µm), the effective photon detection rate $R$ per µm^2^ is calculated as: $R=P/({{{{{{\mathrm{QE}}}}}}} \times A \times {\tau }_{\exp})$ ≈ 6.24 × 10^5^ photons/s per µm^2^. For the ODMR linewidth (see Fig. 1d), we select the narrower one $\triangle v$ = ${\sigma }_{1}$ ~ 6.15 MHz. The averaged contrast C is ~ 0.39%. Therefore, by using these experimental values of $C$ = 0.39%, $\triangle v$ = 6.15 MHz and $R$ = 6.24 × 10^5^/s (per µm^2^), the shot-noise-limited sensitivity is calculated as ${\eta }_{{{{{{\rm{CW}}}}}}-{{{{{\rm{ODMR}}}}}}}=$54.80 µT Hz^−1/2^ (per µm^2^).

## Supplementary information

Supplementary Information

## Data Availability

The datasets generated during and/or analyzed during the current study are available from the corresponding author on reasonable request.
