# Supplementary material for: Sub-micron spin-based magnetic field imaging with an organic light emitting diode
Source: Nat Commun. 2023 Mar 15;14:1441. doi: 10.1038/s41467-023-37090-y (PMC10017713; doi:10.1038/s41467-023-37090-y)
Supplement: Supplementary file 1 — Supplementary Information [file 41467_2023_37090_MOESM1_ESM.pdf]

SUPPLEMENTARY INFORMATION

Sub-micron spin-based magnetic field imaging with an organic light emitting diode

Rugang Geng[1], Adrian Mena[1], William J. Pappas[1], Dane R. McCamey[1]*

[1]ARC Centre of Excellence in Exciton Science, School of Physics, UNSW Sydney, NSW 2052, Australia

*dane.mccamey@unsw.edu.au

Supplementary Method 1: Fabrication of the integrated microwave resonator

Device structure and layer stack developed for this study are based on the requirement to integrate a micron-size OLED with a microwave resonator on the same substrate that provides capability for both EDMR and ODMR measurements at room temperature, good electrical insulation between the OLED from the resonator, and an open ITO surface for the implementation of the micron-size OLED on top of it. To extract the light out of the device from the bottom ITO electrode side, the resonator layer needs to be laterally separated from the ITO electrode. Gold (Au) is an excellent room-temperature conductor and resistant to most acids/chemicals, so we use Au for the resonator layer. The key procedure of integrating the resonator with an OLED on the same ITO-based glass substrate is how to electrically isolate the resonator layer from the two electrode layers of the OLED, which are the bottom ITO electrode as anode, and the top Al electrode as cathode. Here we employ low-temperature atomic-layer-deposition (ALD) method for the insulating layer deposition[1]. Unlike thermal evaporation and e-beam evaporation which have poor step-edge coverage, ALD method is conformal and provides very high-quality insulating layer even with small layer thickness, and this is essential to electrically isolate some areas with sharp edges.

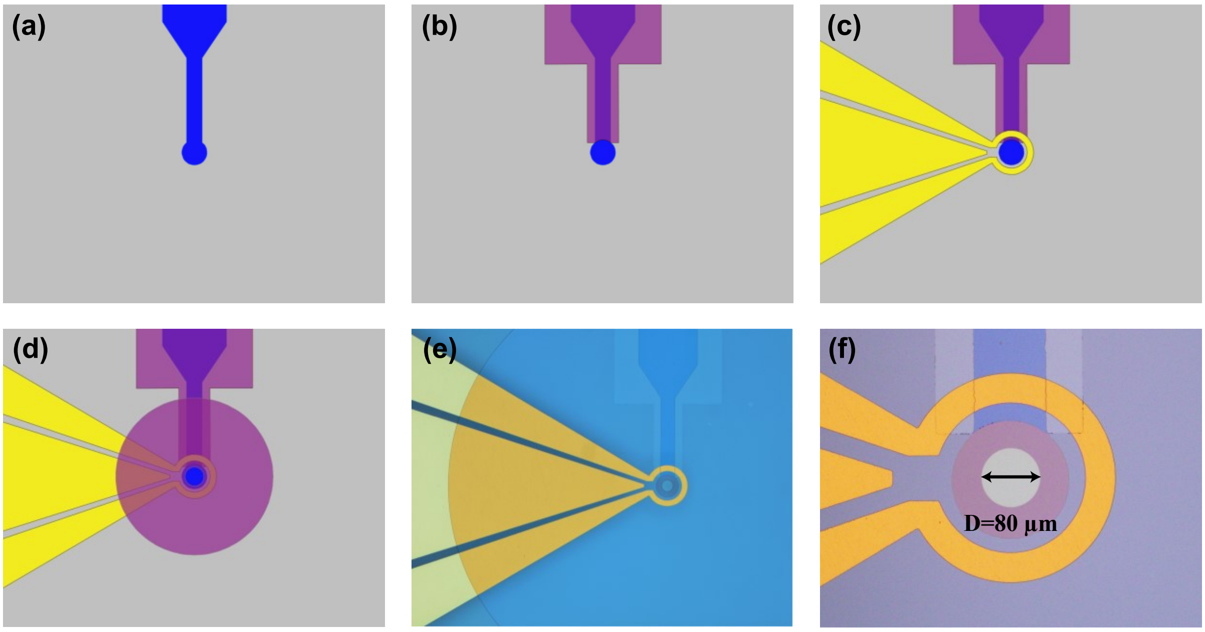

Supplementary Figure 1: (a) Patterned ITO on the glass substrate. The diameter of the electrode end-circle is about 120 µm. **(b)** First insulating layer on top of ITO electrode. **(c)** Microwave omega-shape resonator. The end of ITO electrode sits in the middle of the omega-shape centre, and the intersection between the resonator and the ITO is electrically isolated by the first insulating layer. **(d)** Second insulating layer on top of the Au resonator. The opening hole in the middle of the insulating layer defines the geometry of the active area of the OLED. **(e)** Photograph of the resonator-integrated substrate under 20× magnitude. The right side of the resonator shows more yellowish colour, indicating the area covered by the second insulating layer. Please note that the actual size of the second insulating layer is not scaled with the sketch in (d). **(f)** Zoom-in view of the resonator under 50× magnitude, and it shows a clear active area with diameter of 80 µm in the centre of the resonator. The inner diameter of the omega-shape resonator is ~ 200 µm.

The whole layer structure is as following: glass (0.7 mm) / ITO (120 nm) / Al_2O_3 (45 nm) / Ti (10 nm) / Au (500 nm) / Ti (10nm) / Al_2O_3 (45 nm).

The prepatterned ITO/glass substrates are purchased from Kintec Company (Hong Kong). The ITO substrates are cleaned by standard cleaning procedure and dried out in the vacuum drying oven at 120 °C overnight before usage. The glass substrate dimension is: 30.0 (±0.05) mm × 20.0 (±0.05) mm × 0.7 (±0.01) mm. We note that shadow mask, which will be used for thermal deposition of the top Al electrode of OLED, is precisely cut by laser based on the substrate dimension. The precision of the shadow mask dimension is particularly important for the good alignment between the substrate and the shadow mask, which is the key step in the later OLED fabrication process (see Supplementary Fig. 2).

The photoresist structures for the two insulating layers and the microwave resonator layer are prepared through the standard photolithography process using MA6 system, using negative photoresist nLOF2020 and developer ZA826MIF with optimized parameters. The details of the photolithography steps are as following:

1) Spin nLOF2020 on the substrate at 3000 RPM for 30 s, resulting in photoresist layer thickness of ~2.3 µm
2) Prebake the photoresist at 115 °C for 1 min
3) UV exposure for 4.5 s
4) Post exposure bake (PEB) the photoresist at 115 °C for 1 min
5) Develop in AZ826MIF for 1 min
6) DI water rinse for 20 s, and nitrogen gun dry
7) Further bake at 115 °C for 2 mins to remove any water residue
8) Post plasma cleaning for 10 mins (plasma etching rate ~ 30 nm / per min)

Here we choose Al_2O_3 as the insulating material because of its excellent electrical isolation property, more importantly, its compatibility with the materials and fabrication methods that are used in this work. The breakdown field of Al_2O_3 by ALD at room temperature is about 8 MV/cm (or 0.8V/nm), so 45 nm thickness is thick enough for OLEDs, whose operational voltage is in range of 0 V- 15 V. The ALD system is CNT Savannah S200. The precursors for Al_2O_3 in ALD are water vapor (H_2O) and Trimethylaluminum (TMA). The chamber temperature for the ALD process is set at 120 °C. We note that the chamber temperature cannot be set too high as it would solidate the photoresist and the following lift-off process will become exceedingly difficult. The temperature in principle can be lower such as 80 °C, which will ease the following lift-off process, but the cycle time will increase, and the total deposition time will increase dramatically. There is a trade-off between the deposition temperature and deposition time cost. The total deposition time for 45 nm Al_2O_3 by ALD at 120 °C is about 9.5 hours.

Following up the ALD, the lift-off procedure of Al_2O_3 is carried out by immersing the samples in the N-Methyl-2-pyrrolidone (NMP) bath. To allow the NMP to penetrate the conformal insulating layer and attack the photoresist below quicker, it is necessary to scratch the surface of sample manually and slightly at locations without pattern features. For the bottom insulating layer, we could easily scratch the surface close to the edge of the substrate as there is no patterns underneath; while for the top insulating layer, we employ a Cascade probe station and use the sharp metal probe-tip to crack the photoresist pillar inside the resonator gently top downwards. After the scratch, samples are immerged in the NMP bath on hotplate at 100 °C in a fume cupboard, until the lift-off procedure is completed.

For the resonator layer deposition, the substrates with prepatterned photoresist structure are transferred to a thermal evaporation chamber (Jurt J. Lesker) for the metal deposition. The vacuum condition is of ~10^{-6} mbar, and the layers stack is Ti (10 nm) / Au (500 nm) / Ti (10nm).

The first 10 nm Ti layer is deposited as adhesion layer for the following Au deposition onto the glass surface. For the Au layer deposition, the first 100 nm is deposited with a low rate of 0.5 A/s, to minimize the heating effects on the prepatterned photoresist structure, such as deforming or softening; the next 400 nm is deposited with a high rate of 2 A/s for time saving. The second 10 nm Ti layer is deposited as another adhesion layer for the spin-coating photoresist in the following photolithography procedure. Standard lift-off is followed in the NMP bath at 100 °C.

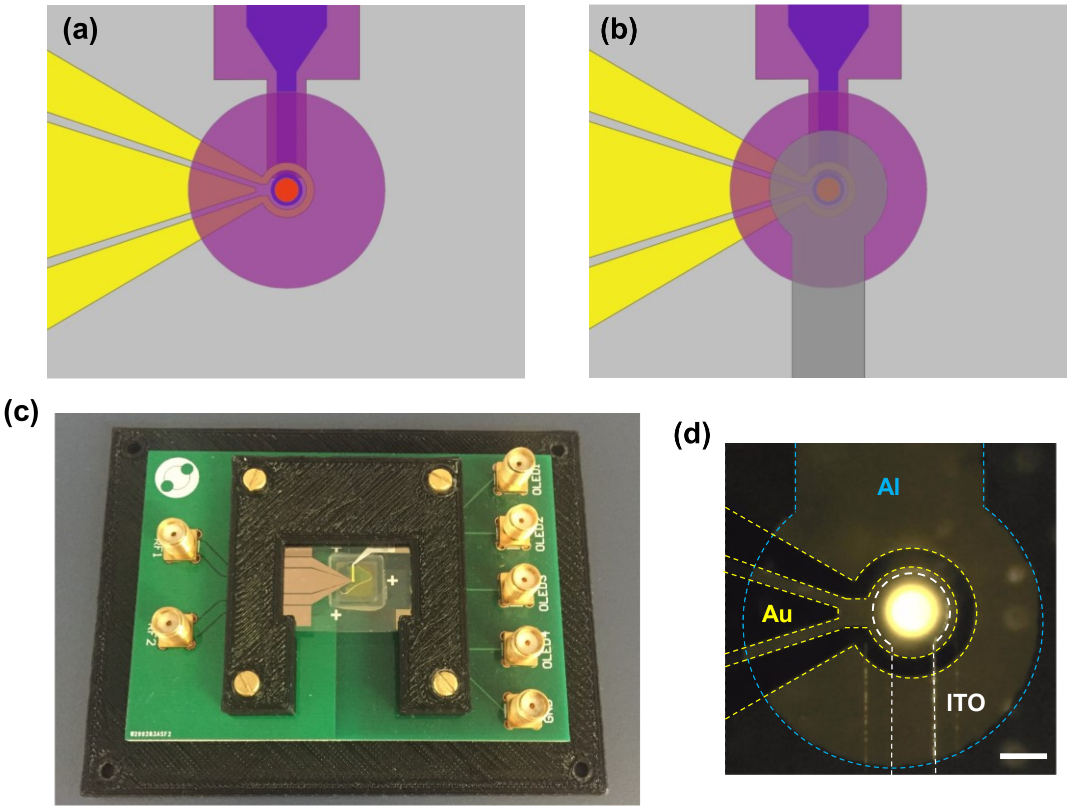

Supplementary Figure 2: sketch of the device fabrication **(a)** where a micron-size OLED is fabricated inside the active area ($D \sim 80$ μm) and **(b)** the top Al electrode is deposited by using a well aligned shadow mask. **(c)** Photograph of the PCB platform for the device mounting and electrical connection. The device is mounted onto the PCB via a 3D printed plastic lid. The device is electrically connected to the PCB via pogo pins (both AC for the resonator and DC for the OLED). The OLED is encapsulated by using a square glass coverslip (10 mm × 10 mm) with a cavity (300 μm depth) in it to avoid physical contact with the top Al electrode. **(d)** Photograph of the device under operation. Profiles of the bottom ITO electrode, the top Al electrode, and the Au resonator are highlighted using dashed lines. There is a small offset of the top Al electrode from the centre, which is due to the manual alignment of the shadow mask through the OLED fabrication procedure. The scale bar in (d) is 100 μm.

Supplementary Method 2: Background noise and RF power broadening

There is a "wobbling" feature noise in the raw magnetic resonant spectrum with sweeping the microwave frequency. We suspect that the noise origins from the microwave component in the experimental setup as we see similar "wobbling" feature in the S_{11} curve of the SMA cable, which might be due to the mismatch of 50 Ω impedance of the RF output, resulting in some end-reflection of the signal in the cable. Such noise is then transmitted and modified through the PCB and the resonator, and eventually coupled to the OLED, resulting in those "wobbling" feature noise in the final EDMR spectrum (Supplementary Fig. 4) contributing to the overall background noise. In addition, the amplitude of the noise is not a constant, but varies at different microwave frequency (Supplementary Fig. 3). Such baseline noise can be measured separately and then subtracted from the raw experimental data (Supplementary Fig. 4). We note that such impedance mismatch and relevant noises due to possible electrical coupling between the RF source and the OLED could be potentially minimized by optimizing the device architecture, such as the geometry of the resonator and the spatial distance and relative orientation between the resonator and the OLED.

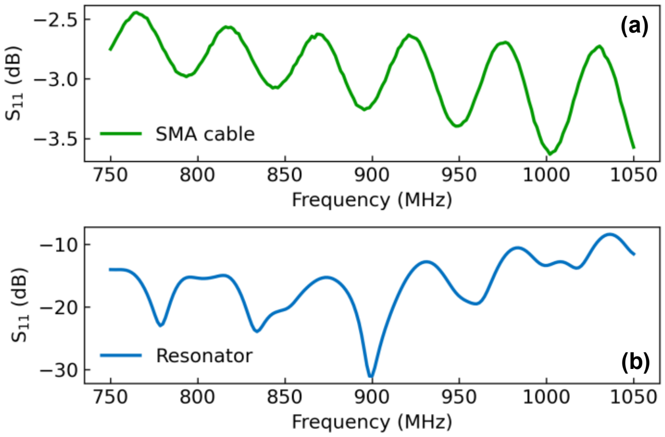

Supplementary Figure 3: S_{11} curve of **(a)** SMA cable itself, which is connected to the microwave source directly, and **(b)** the microwave resonator connected to the microwave source through the PCB, showing a resonant frequency around 900 MHz.

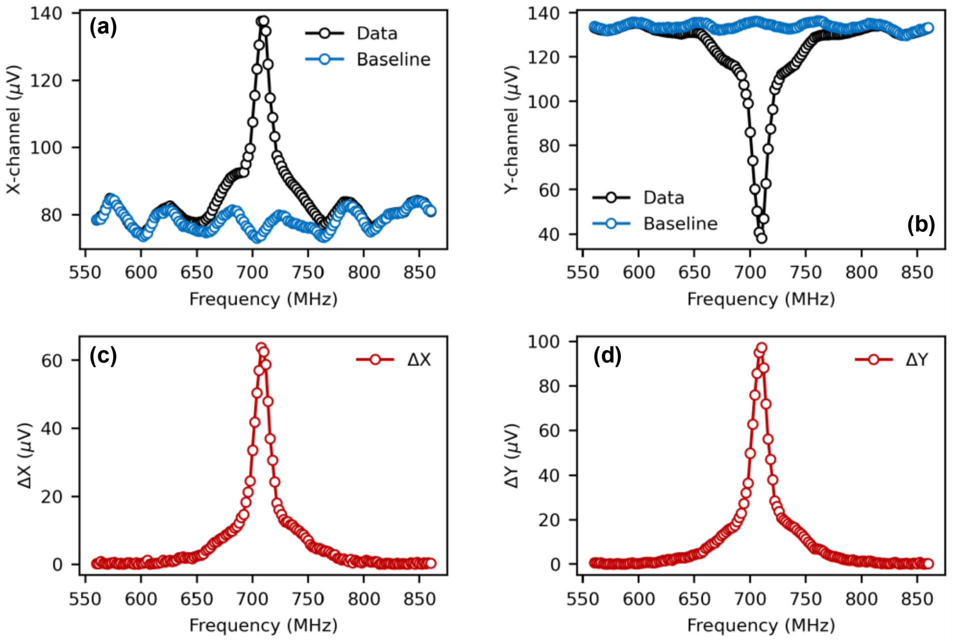

Supplementary Figure 4: Raw signal and background noise in **(a)** X-channel and **(b)** Y-channel of the lock-in detection where the microwave frequency is swept. The EDMR signal in **(c)** X-channel and **(d)** Y-channel after the background noise is subtracted. The final EDMR signal is given by $\Delta R = \sqrt{(\Delta X)^2 + (\Delta Y)^2}$ with negative sign[2,3].

We also investigate the effect of the RF power broadening on the magnetic resonance spectrum. Supplementary Fig. 5(a) shows that the EDMR signal (signal sign is negative) amplitude monotonically increases when the RF power increases (equivalently B_1 field strength increases). This is consistent with previously literature reports[3], confirming that the RF power (~5 dBm) used in the experiment is indeed in the low RF driving power regime ($B_1 << B_{\mathrm{hyp}}$). In this low driving power regime, the power broadening effect is negligible, and the magnetic resonant spectrum (both EDMR and ODMR) can be decoupled into two Gaussian functions with equal g-factor of ~2.0026. In addition, all the EDMR spectrums with different RF power are well fit using two Gaussian functions and more importantly the center of the peak remains the same position, indicating that the effect of RF power on field detection accuracy is also negligible.

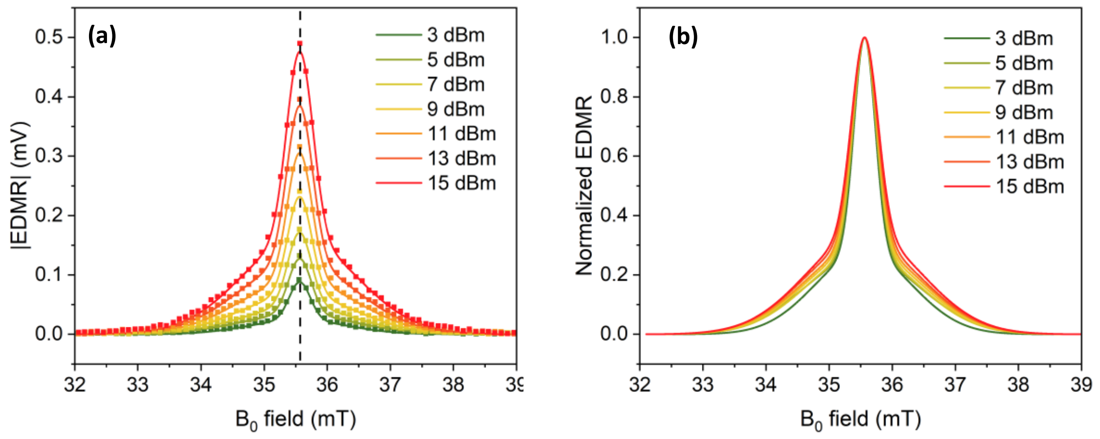

Supplementary Figure 5: (a) EDMR spectrums with different RF power output from the signal generator. The dots are experimental data, and the solid curves are the fittings described by double Gaussian functions with equal g-factors. **(b)** Normalized fitting curves with different RF power.

Supplementary Method 3: Angle dependent EDMR

To verify that the magnetic resonance condition is independent of the orientation of the external magnetic field B_0 in our device, a separate angle dependent EDMR measurement is carried out. As sketched in Supplementary Fig. 6(a), the device is mounted on a rotation stage sitting between two electromagnet poles. Instead of changing the orientation of the field B_0, the orientation of microwave field B_1 is changed equivalently. When the device rotates, the orientation of the microwave field B_1 rotates in the horizontal $x - y$ plane, labelled by angle θ as shown in Supplementary Fig. 6(b). We find that: (1) the amplitude of the EDMR spectrum peak, which corresponds to the maximum change of the EDMR signal, is proportional to the projection of the B_1 field along the orthogonal direction of B_0, and the relationship between the amplitude and the rotation angle can be well fit by a sine wave function; (2) the resonant frequency of the EDMR spectrum peak (f), which corresponds to the external magnetic field B_0 ($f = \gamma B_0$,), remains the same with 99.94% confidence.

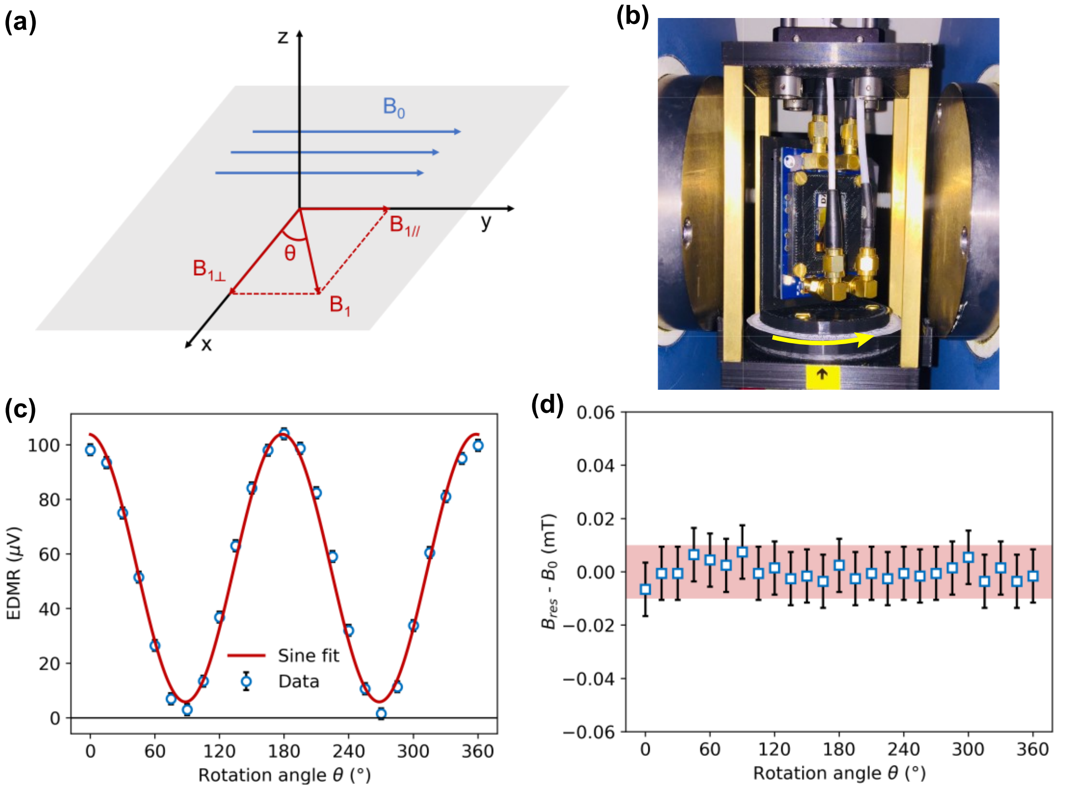

Supplementary Figure 6: (a) Rotation scheme where B_0 is the static magnetic field generated by the two electromagnet poles with fixed orientation, and B_1 is the oscillating field generated by the integrated microwave resonator on the device. The orientation of field B_1 rotates in the horizontal $x - y$ plane along with the stage rotating. **(b)** Photograph of the experimental setup. Device is mounted on PCB vertically, and the PCB is mounted on a rotation stage. **(c)** Amplitude of the resonant peak of the EDMR spectrum as a function of the rotation angle θ, where the frequency of the microwave field B_1 is fixed at 700 MHz and the magnetic field B_0 is swept. **(d)** The difference of resonant B_0 field between the experimental value extracted from the EDMR spectrum and the theoretical value calculated by $B_0 = f/\gamma$, where f = 700 MHz and γ = 28.03 (GHz/T). The error bar corresponds to the magnetic field step accuracy (0.02 mT) in this measurement.

We also observed an exceedingly small EDMR signal at θ = 90° and θ = 270°, which is attributed to the spatial variation of the microwave field. The distance between the microwave resonator and the OLED is much smaller than the wavelength of the microwave radiation, hence the OLED is in the near-field region of the B_1 field. As a result, the spatial orientation of the B_1 field is determined by the dimensions of the resonator itself and the surrounding conductors, and the orientation varies slightly in the OLED region. Therefore, there is always a small in-plane projection ($B_{1//}$) of B_1 field during the whole rotation, and it plays a dominant role in the non-zero EMDR spectrums at θ = 90° and θ = 270°.

The small variation of resonant B_0 field value origins from the following aspects: (1) The influence of the SMA connectors on the PCB. We find that those SMA connectors used in this work show weak paramagnetic behaviour under large external magnetic field, and it leads to a very weak disturbance on the Gauss probe reading during the rotation. The disturbance becomes noticeable (at 0.1 mT scale) at some angles (60° to 120°) where the SMA connectors are closest to the Gauss probe. We note that such disturbance has been removed in Supplementary Fig. 6(d) through an independent and careful calibration process. (2) The finite step size of the sweeping magnetic field, which results in an uncertainty of the B_0 value extraction. (3) The uniformity of the static magnetic field B_0 between the two electromagnet poles. The uniformity of the field depends on the dimensions of the two poles, the gap between them, and the spatial location. In practice, the magnetic field detected by the Gauss probe is always slightly different from the actual field the device experiences, and such difference may even vary during the rotation as the rotation setup is not perfectly aligned with the magnet.

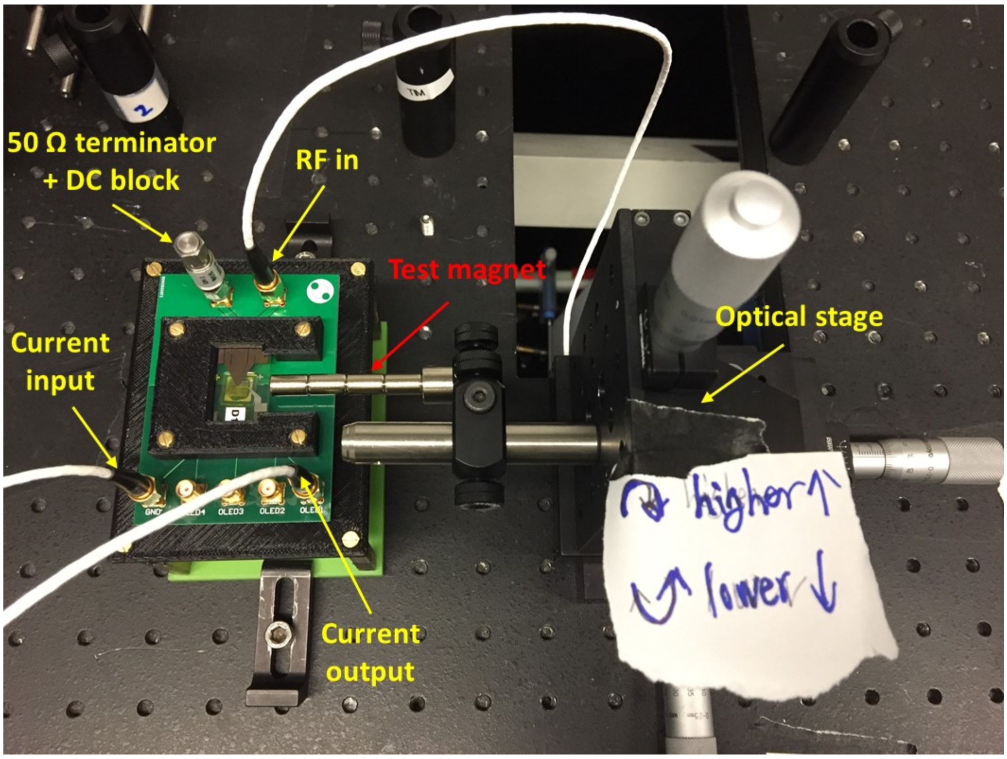

Supplementary Figure 7: Setup for frequency-swept-based EDMR measurement.

Supplementary Method 4: Simulation of the magnetic field

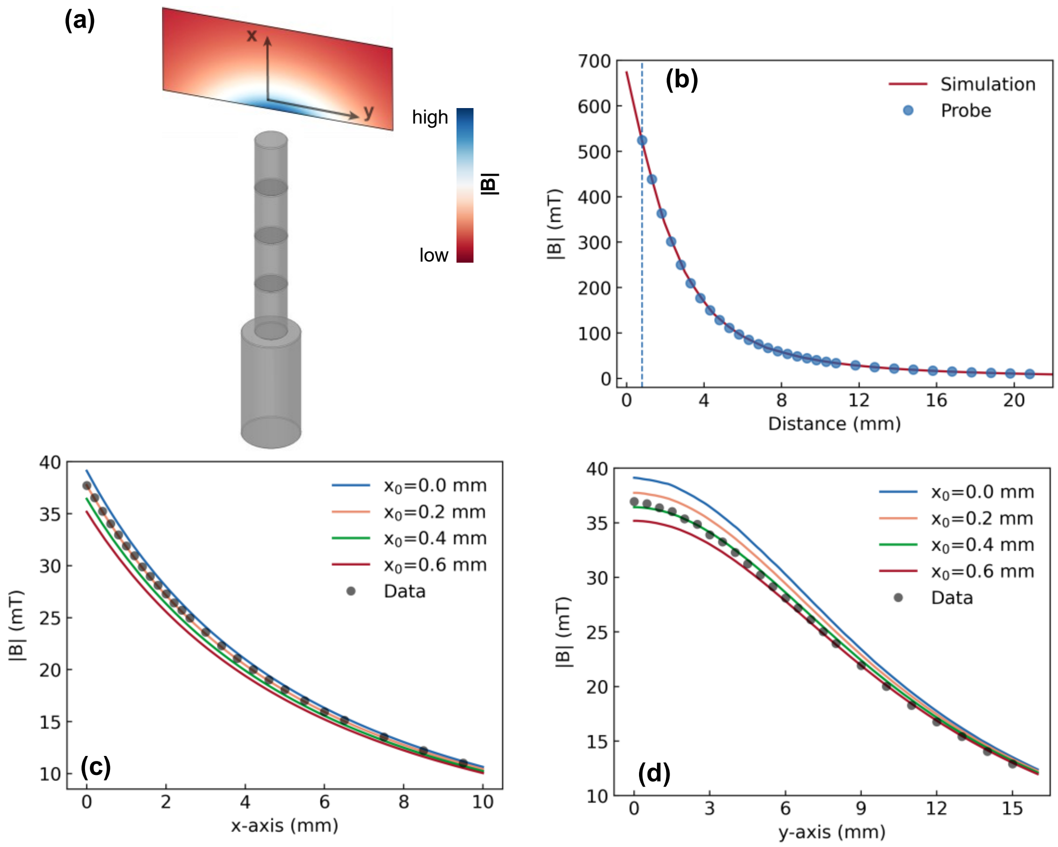

Supplementary Figure 8: (a) Magnetic field simulation of the test magnets using ANSYS Electronics (Maxwell 3D Design with Magnetostatic Solution). The 2D map represents the spatial distribution of the strength of the static magnetic field generated by the magnet in a region of 14.0 mm × 36.0 mm in the $x - y$ plane with a gap distance of d = 10.0 mm from the top surface of the magnet. Please note that the test magnets are not drawn to scale. Dimensions and material properties of these cylindrical magnets are summarized in Supplementary Table 1. **(b)** Comparison of the simulated magnetic field and the experimentally measured field using Gauss probe. The strength of the magnetic field here is a function of the movement distance along the z-direction from the top surface of the magnet. We note that the total thickness of the Hall probe is about 1.6 mm, therefore the starting position for the Hall probe measurement is about 0.8 mm (half of the probe thickness) as labelled out by the dash line, where we positioned the probe right adjacent to the magnet. The similarity between the Hall probed field and the simulation field is 98.5 %. Comparison of experimentally measured field (grey dots) and the simulated magnetic field (colour curves) with various starting position x_0, as a function of the movement distance along x-direction in **(c)** and along y-direction in **(d)**, respectively. We note that the $x - y$ coordinates in (c) and (d) is a local frame within the 2D map plane, which is labelled out separately in (a). Similarity between the measured field and the simulation field is 99.8 % (x_0 = 0.2 mm) in (c), and 98.6 % (x_0 = 0.4 mm) in (d). The similarity calculation formula is listed as below.

	Diameter (mm)	Length (mm)	Edge radius (mm)	Materials
**Cylinder 1**	7.0	12.0	0.2	N48
**Cylinder 2**	12.7	25.4	0.2	N45

Supplementary Table 1: Parameters of the magnet cylinders.

We note that the parameters of diameter, length and material property are obtained directly from the product datasheet, and the edge radius is estimated based on our own measurements. The edge radius here refers to the smooth curvature of the surface edge of the magnet cylinder. In addition, the influence of the edge radius on the simulation field distribution is investigated by comparing simulation with edge radius 0.2 mm and simulation with edge radius 0.0 mm. We find: (1) for the far-field region (d > 8.0 mm), the difference of the field between two cases, both amplitude and direction, is negligibly small; (2) for the near-field region (d < 5.0 mm around the edge area), the difference is still quite small but not negligible. Therefore, in the Main Fig. 2 where the distance d > 10 mm, simulation field remains the same regardless the estimated value of the edge radius.

- Definition of similarity: $s = 1 - \frac{1}{N}\sum_{i=1}^{N}\frac{|p(x_i) - q(x_i)|}{[p(x_i) + q(x_i)]/2}$, where $p(x_i)$ and $q(x_i)$ are two independent data sets as a function of variable x_i .

Supplementary Method 5: Standard error calculation

- **Standard error in the local region**

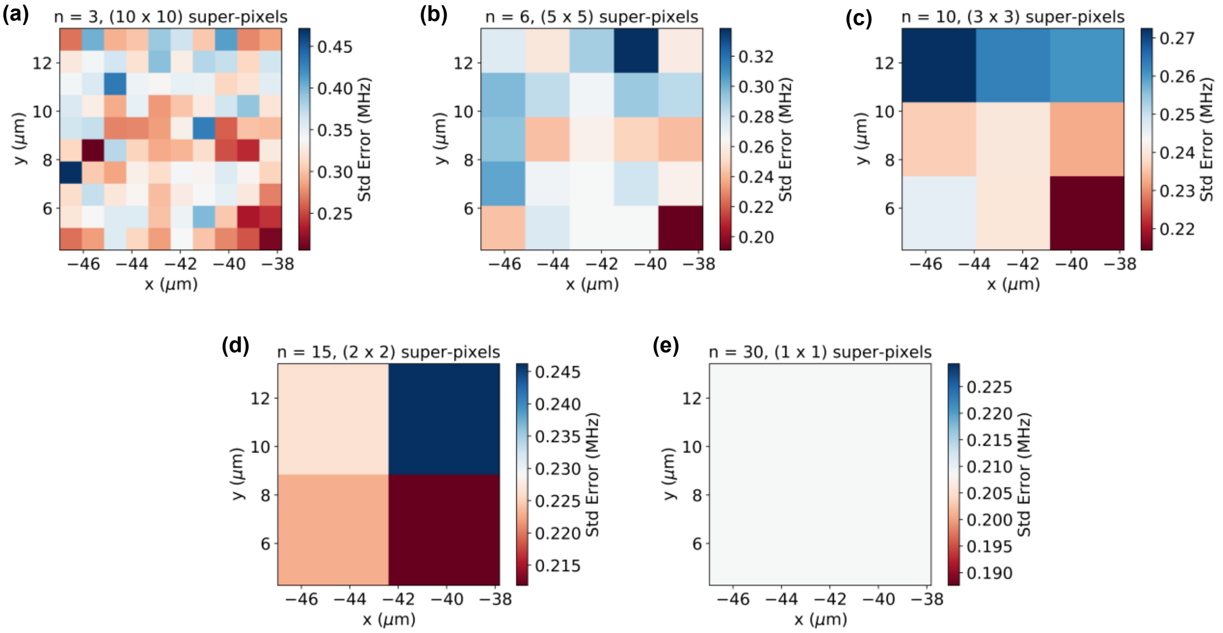

Supplementary Figure 9: standard error of the fit of the resonant peak frequency with a variety of binning size in the local region. **(a)** binning size $n = 3$, **(b)** binning size $n = 6$, **(c)** binning size $n = 10$, **(d)** binning size $n = 15$, and **(e)** binning size $n = 30$.

- **Standard error in the OLED region and diffusion region**

Figure 9(a) shows the spatial distribution of the standard error (SE) of the resonant peak frequency of the ODMR spectrums. The 2D map demonstrates three distinguishable regions: (1) the central region with $R < R_2$, where the SE is clearly larger than the surrounding regions. The reason of the large SE in this region is because of the electrical coupling between the resonator and the device electrodes. We suspect that the 'wobbling' feature noise from the resonator (see Supplementary Fig. 3) is encoded into the device where an electrical coupling between the ITO and Al electrodes is induced. Such electrical coupling is eventually transmitted to the device output (both current and the EL), reducing the overall SNR. (2) the ring region with $R_2 < R < R_3$, where the SE is the smallest. The EL emission in this ring region is due to the high hole mobility in the PEDOT:PSS layer. In specific, holes are injected from ITO electrode into PEDOT:PSS layer through the defined area (R_{OLED} = 40 μm), then diffuse in the PEDOT:PSS layer along the in-plane direction outwards. Under the bias voltage, these diffusing holes are gradually injected into the emitting layer along the diffusion path, eventually forming excitons through combing with the electrons which are injected from the top Al electrode. As this is the diffusion region, the noise caused by the electrical coupling between the two electrodes is much weaker compared to the central region. Therefore, the SE in this ring region is smaller than the central region. (3) the edge region with $R > R_3$, where the SE is large compared to ring region though it is also the diffusion region. The reason is because the EL signal is much weaker in this edge region, therefore the over SNR is much smaller.

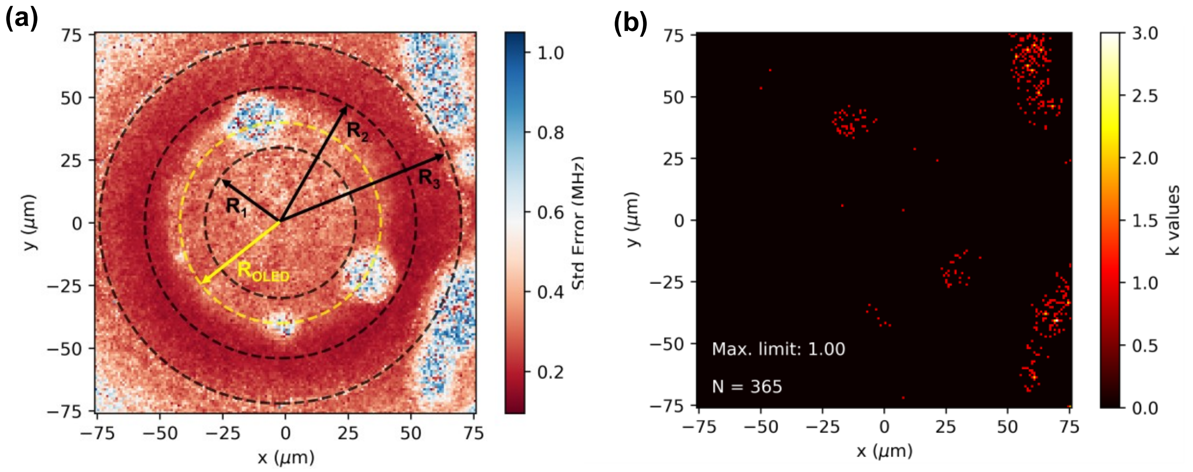

Supplementary Figure 10: (a) 2D map of the standard error of the resonant peak frequency fit with binning size n =3 (the whole region contains of 166 × 166 super-pixels). The OLED region refers to the region with $R < R_1$ and the diffusion region refers to the region with $R_2 < R < R_3$. The yellow dash circle with radius R_{OLED} indicates the edge of the OLED area defined by the photolithography process. Their values are: R_1= 30 µm, R_2 =54 µm, R_3 = 72 µm, R_{OLED} = 40 µm. **(b)** Spatial distribution of outliers which are defined as the points whose value is out of the field range of (754 MHz, 771 MHz). The actual values of the resonant peak frequency and the standard error of those outliers are interpolated by adjacent points, the distance of which is indicated by k value. The selection of the values of those radiuses is to avoid the outliers while covering as many points as possible in each region. Those outliers are caused by defects during photolithography process and device degradation during the measurement.

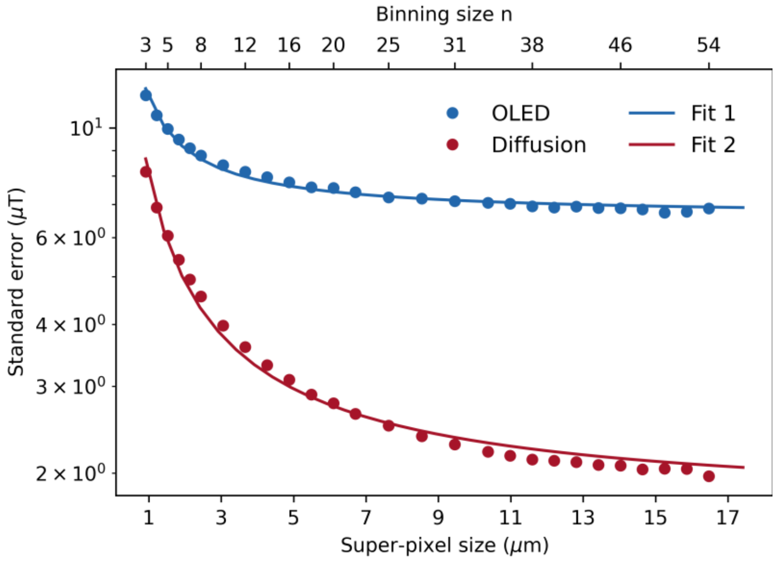

Supplementary Figure 11: Standard error (SE) of double Gaussian fits of the spatially resolved ODMR spectrums with different binning sizes.

Supplementary Method 6: Sensitivity calculation of the spatially resolved ODMR

- **Magnetic field sensitivity**

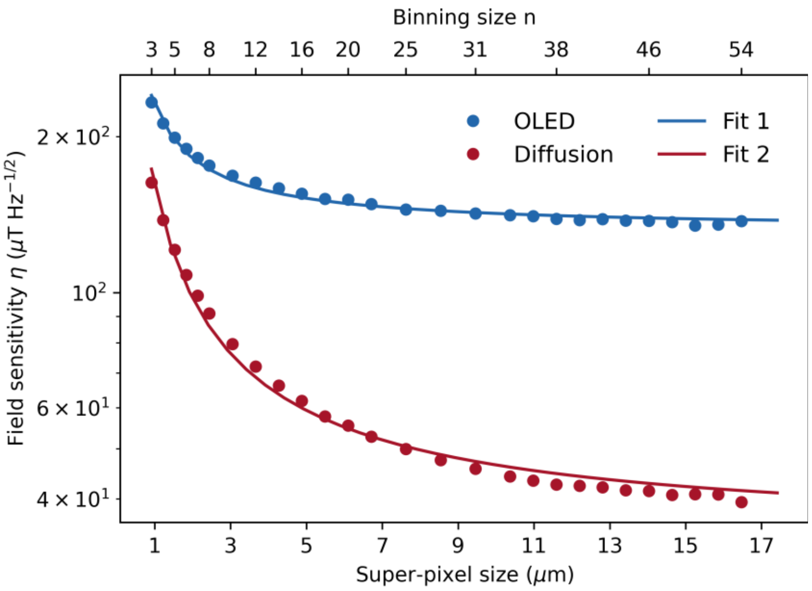

Supplementary Figure 12: Magnetic field sensitivity of the spatially resolved ODMR as a function of super-pixel size in both OLED and diffusion regions. See more details about the region definition in Supplementary Figure 10. The data set can be fit by using function $y = a/x + b$. The fit parameters are (1) for the OLED region: $a = 98.81(\pm 2.12)$, $b = 132.33(\pm 0.76)$; (2) for the diffusion region: $a = 127.55(\pm 2.44)$, $b = 33.77(\pm 0.87)$. The unit for parameter a and b is $\mu T \cdot Hz^{-1/2} \cdot \mu m$ and $\mu T \cdot Hz^{-1/2}$, respectively.

- **Magnetic field gradient sensitivity**

The magnetic field gradient sensitivity is calculated from the uncertainty of the field gradient, which is given by $G = \Delta B/\Delta x$, where $\Delta B = B(x_2) - B(x_1)$ is the measured magnetic field difference between two super-pixels located at x_1 and x_2, and $\Delta x = x_2 - x_1$ is the distance between them (refers to the inset in Main Figure 4). Based on the error propagation, the minimum detectable gradient $\delta G_{\min}$ is given as following:

$$\frac{(\delta G_{\min})^2}{G^2} = \frac{[\delta(\Delta B)_{\min}]^2}{(\Delta B)^2} + \frac{[\delta(\Delta x)_{\min}]^2}{(\Delta x)^2} \qquad (1)$$

Because of $\Delta B = B(x_2) - B(x_1)$, so $[\delta(\Delta B)_{\min}]^2 = [\delta B(x_2)_{\min}]^2 + [\delta B(x_1)_{\min}]^2$, where $\delta B(x_i)_{\min}$ $(i = 1,2)$ is the uncertainty of the magnetic field (or minimum detestable magnetic field difference). Here we assume that uncertainty of the magnetic field is location independent or ideally the same across the whole device, $\delta B(x_2) = \delta B(x_1) = \delta B$, therefore we have the following result:

$$\delta(\Delta B)_{\min} = \sqrt{2} \times \delta B_{\min} \qquad (2)$$

Similarly, we can have the following result:

$$\delta(\Delta x)_{\min} = \sqrt{2} \times \delta x_{\min} \qquad (3)$$

where $\delta x_{\min}$ is the uncertainty of the distance measurement and we assume $\delta x_{\min}$ is location independent as well.

For a digital distance measurement, the minimum uncertainty is equal to the super-pixel width (see the inset in figure 4 in the main content),

$$\delta x_{\min} = w \qquad (4)$$

As the whole measurement system is fixed firmly on the optical table, and no relative movement between the camera and device has been observed in a similar setup from our previous work[4], we believe that the actual uncertainty of the distance measurement caused by vibration and relative displacement is negligibly small compared to w.

By plugging equations (2-4) into equation (1) and using $G = \Delta B / \Delta x$, we can get:

$$\frac{(\delta G_{\min})^2}{G^2} = \frac{2(\delta B_{\min})^2}{(\Delta B)^2} + \frac{2(\delta x_{\min})^2}{(\Delta x)^2} \qquad (5)$$

thus

$$(\delta G_{\min})^2 = G^2 \left[\frac{2(\delta B_{\min})^2}{(\Delta B)^2} + \frac{2(\delta x_{\min})^2}{(\Delta x)^2} \right]$$

$$= G^2 \frac{2(\delta B_{\min})^2}{(\Delta B)^2} + G^2 \frac{2(\delta x_{\min})^2}{(\Delta x)^2}$$

$$= \frac{(\Delta B)^2}{(\Delta x)^2} \frac{2(\delta B_{\min})^2}{(\Delta B)^2} + G^2 \frac{2(\delta x_{\min})^2}{(\Delta x)^2}$$

$$= \frac{2(\delta B_{\min})^2}{(\Delta x)^2} + \frac{2G^2(\delta x_{\min})^2}{(\Delta x)^2} \qquad (6)$$

Square root both sides of equation (6), we can get:

$$\delta G_{\min} = \sqrt{2} \frac{\delta B_{\min}}{\Delta x} [1 + (\frac{\delta x_{\min}}{\delta B_{\min}})^2 G^2]^{1/2} \qquad (7)$$

Equation (7) can be rewritten as below:

$$\delta G_{\min} = \sqrt{2} \frac{\delta B_{\min}}{\Delta x} [1 + (\frac{\delta x_{\min}}{\delta B_{\min}})^2 (\frac{\Delta B}{\Delta x})^2]^{1/2} \qquad (8\text{-}1)$$

$$\delta G_{\min} = \sqrt{2} \frac{\delta B_{\min}(w)}{\Delta x} \{1 + [\frac{w}{\delta B_{\min}(w)}]^2 (\frac{\Delta B}{\Delta x})^2\}^{1/2} \qquad (8\text{-}2)$$

In the above equation (8-1), $\delta B_{\min}$ is the minimum detectible magnetic field difference (or uncertainty of the magnetic field as mentioned above), and it depends on the super-pixel size w (or binning size n) as shown in Supplementary Figure 11; $\delta x = w$ is the uncertainty of the distance measurement as shown in equation (4). Equation (8-1) can also be rewritten as equation (8-2) which shows all the independent variables: $\delta B_{\min}$, w, Δx, and ΔB.

According to equation (8-2), $\delta G_{\min}$ is a monotonic increasing function of variable ΔB with fixed value of $\delta B_{\min}$, w and Δx. Therefore, the minimum detectable gradient $\delta G_{\min}$ can be further decreased by choosing the minimum distinguishable field difference between two neighbouring points $\delta(\Delta B)_{\min} = \sqrt{2} \times \delta B_{\min}$ as shown in equation (2). By plugging $(\Delta B)_{\min} = \sqrt{2} \times \delta B$ into equation (6-2), we can get δG as below:

$$\delta G_{\min} = \sqrt{2} \frac{\delta B_{\min}(w)}{\Delta x} [1 + 2(\frac{w}{\Delta x})^2]^{1/2} \quad (\Delta x \geq w) \qquad (9)$$

Finally, considering the measurement time, the magnetic field gradient sensitivity η_G is given as below:

$$\eta_G = (\delta G_{\min}) \times \sqrt{T}$$

$$= \sqrt{2T} \frac{\delta B_{\min}(w)}{\Delta x} [1 + 2(\frac{w}{\Delta x})^2]^{1/2} \qquad (10)$$

Please note that all the uncertainties (or errors) in equation (10) should be relatively small to justify the error propagation analysis above.

References:

S1. Biercuk, M. J., Monsma, D. J., Marcus, C. M., Becker, J. S. & Gordon, R. G. Low-temperature atomic-layer-deposition lift-off method for microelectronic and nanoelectronic applications. *Appl. Phys. Lett.* **83**, 2405-2407 (2003).

S2. Jamali, S., Joshi, G., Malissa, H., Lupton, J. M. & Boehme, C. Monolithic OLED-Microwire Devices for Ultrastrong Magnetic Resonant Excitation. *Nano Lett.* **17**, 4648-4653 (2017).

S3. Waters, D. P. *et al.* The spin-Dicke effect in OLED magnetoresistance. *Nat. Phys.* **11**, 910-914 (2015).

S4. Pappas, W. J. *et al.* Resolving the Spatial Variation and Correlation of Hyperfine Spin Properties in Organic Light-Emitting Diodes. *Adv. Mater.* **34**, 2104186 (2022).
